# Potential of Marine Biomolecules: Advances in Extraction and Applications of Proteins, Polysaccharides, and Antioxidant Compounds

**DOI:** 10.3390/foods14152555

**Published:** 2025-07-22

**Authors:** Gabriela Sousa, Suzana Ferreira-Dias, Carla Tecelão, Vítor D. Alves

**Affiliations:** 1 Instituto Superior de Agronomia, LEAF—Linking Landscape, Environment, Agriculture and Food Research Centre, Universidade de Lisboa, 1349-017 Lisboa, Portugal; gabriela.spsousa@gmail.com (G.S.); vitoralves@isa.ulisboa.pt (V.D.A.); 2 MARE—Marine and Environmental Sciences Centre, ARNET—Aquatic Research Network, Politécnico de Leiria, 2520-641 Peniche, Portugal; carla.tecelao@ipleiria.pt; 3 Laboratório de Estudos Técnicos, Instituto Superior de Agronomia, Universidade de Lisboa, 1349-017 Lisboa, Portugal; 4 Associate Laboratory TERRA, Instituto Superior de Agronomia, Universidade de Lisboa, 1349-017 Lisboa, Portugal

**Keywords:** marine macroalgae, biopolymers, antioxidant molecules, fish by-products

## Abstract

Oceans are increasingly viewed as a new frontier for economic development, contributing to the bridge between food industry, sea bioeconomy, and health. Nowadays, oceans are under attention as a strategy for creating opportunities and driving innovation, and their vital importance will become even more evident in the years to come. Therefore, it is crucial to study oceans under a holistic approach, taking the maximum value of their abundant resources in a sustainable way. As such, blue bioeconomy is the path forward, since it is a development strategy that meets the economic potential without compromising the environmental health. A special look needs to be taken at the underutilized resources and by-products, which hold unexploited value. For instance, green macroalgae are widespread marine macroalgae that lack industry applications, despite being rich in biopolymers (polysaccharides) and antioxidants. Moreover, fish by-products are also rich sources of biopolymers, mostly proteins. Thus, among other potential uses, raw materials could be explored to produce functional edible coatings under a blue bioeconomy approach. A detailed characterization of raw materials is the first step for the development of value-added products. These topics will be addressed in this review.

## 1. Introduction

Oceans cover 70% of the Earth and are essential for human survival, playing a pivotal role in diverse aspects that sustain life. They produce over half of the oxygen we breathe. Furthermore, they absorb and store large amounts of carbon dioxide and are critical carbon sinkers [1,2]. In addition, oceans absorb excess heat, regulate the climate, influence weather patterns, and maintain the delicate balance of temperature that makes Earth habitable [3]. Simultaneously, oceans are the planet’s largest life-support system, constituting a vast reservoir of biodiversity with approximately 250,000 marine species [4,5].

However, despite their recognized vastness and critical importance, uncontrolled human population growth and its consequent high activity at the sea, along with pollution, are damaging the oceans, highlighting the fragility of aquatic ecosystems [1].

Over the last century, the world population has grown exponentially, and it is expected to keep growing, reaching between 9 and 11 billion by 2050 [6]. This growth is accompanied by a drastic demand for plastic products, a ubiquitous commodity of modern society [7,8]. Conventional plastic products are derived from petroleum and can be easily processed at low cost with the desired functional properties. Their high convenience resulted in a drastic increase in plastic production, from 2.3 million tons in 1950 to 448 million tons in 2015 [4]. This amount is expected to keep growing and to double by 2050 [4,9]. Nevertheless, plastics have limitations in biodegradability and reusability, and their recycling rates are declining, which leads to an accumulation cycle. Consequently, an enormous amount of plastic trash is found in several places around the world [10,11]. Plastic litter accumulates in natural freshwater ecosystems like rivers and streams or, alternatively, it can be leached into the ground water. In most scenarios, plastic ends up in the oceans, turning them into floating islands with more than 260,000 tons of plastic, such as the Great Pacific Garbage Patch [12,13]. Once on the surface of seawater, plastic litter reduces the level of dissolved oxygen and light penetration, causing a decrease in biodiversity. Additionally, as a result of environmental factors such as physical abrasion, exposure to UV radiation, salinity, tidal hydrodynamics, and weather, plastic fragmentation occurs. Hence, increasingly smaller pieces of plastic are obtained and converted into microplastics, which affect the entire food chain. These microplastics negatively impact reproduction, cause genetic mutations, and lead to physiological changes in phytoplankton, zooplankton, fish, and large marine organisms [14]. Furthermore, plastics can contribute to the distribution of non-native invasive species and accumulate toxic chemical compounds by adsorption [15,16].

Simultaneously, human activities in the sea, such as overfishing, destructive fishing practices, and coastal development, are increasing the atmospheric concentration of carbon dioxide, which leads to warmer and more acidic ocean water, resulting, once more, in biodiversity losses. Fisheries and seafood industries also negatively contribute to this urgent problem by discarding countless residues back in the ocean. Human behavior is threatening the ocean balance and, consequently, risking life on Earth [17,18,19,20].

Nowadays, people are becoming aware of the impacts of their actions and are starting to take a position to support the safe, conscious, and effective use of oceans and their resources while protecting the environment. In this context, the blue bioeconomy concept has emerged and is receiving unprecedented attention from a diversity of actors and industries that look at oceans as a promising engine for economic growth [19,21].

Oceans are seen as a new economic frontier, with great potential for development in their vast spectrum of activities, including marine renewable energy (offshore wind), port activities, shipbuilding and repair, maritime transport and coastal tourism, fishing and aquaculture, desalinization, extraction of marine minerals, maritime defence, security, and surveillance. These and other marine activities are expanding rapidly, driven by population and economic growth, trade, rising income levels, and technological advances [5,19]. The extension of these activities represents an opportunity for employment and the development of coastal communities and countries with significant coastal areas, such as Portugal. Portugal, located in the southwest of Europe, is bordered by the Atlantic Ocean in an estimated extension of 987 km and has a long maritime tradition. In terms of gross value added, Portugal’s blue economy accounts for 3.9%, exceeding the contribution of traditional sectors such as agriculture (2.4%) and energy (3.6%), and still holds significant potential for further development [5,17,22,23].

The expansion of ocean activities will demand responsible and sustainable approaches, since oceans play a role in many of the world’s challenges in the decades to come, including global food security, provision of energy, natural resources, climate change, and improved medical care. Therefore, blue bioeconomy is an emergent development approach that considers sustainable and inclusive development, promotion of ecosystem-based climate change mitigation, protection of coasts and oceans, provision of water, energy and food security, protection of health, livelihoods, and welfare of the people in the coastal areas, and a reduction in environmental risks and ecological scarcities [19,24].

In this scope, the first step to successfully design an ocean development project is understanding each problem, aiming to find targeted solutions to it. Following this, about 25–35% of fish meal and oil is already produced from fish by-products, which include fish frames, heads, scales, skin, viscera, and bones. Nonetheless, 60% of the fishing industry by-products are dumped back into the ocean. A proper use of these residues would allow for the recovery of gelatine and other high-value biomolecules, which could be valorized and commercialized, contributing to the reduction of ocean contamination while improving the management of marine resources and increasing the sector’s competitiveness [1,18,25]. For instance, fish viscera are rich in proteins, collagen peptides, lipids (omega 3 polyunsaturated fatty acids), polysaccharides, minerals (e.g., calcium), and bioactive compounds, presenting great potential for the development of high-value products not only in nutraceuticals but also in pharmaceutical areas. As such, these marine by-products hold untapped potential for sustainable development while contributing to waste reduction and eco-innovation [26].

**Figure 1:**
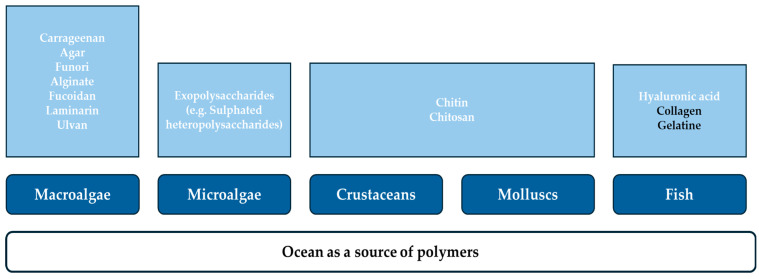
Ocean biopolymers and their sources—polysaccharides (white) and proteins (black).

To overcome the accumulation of plastic, one of the major environmental global issues, biodegradable and renewable alternatives from biological resources are attracting the interest of academics and industries. New materials based on biopolymers, such as polysaccharides (e.g., starch, chitosan, cellulose, alginate, pectin, and carrageenan), proteins (e.g., gelatine, whey protein, and casein), and lipids (e.g., beeswax and fatty acid esters), might provide a sustainable solution to replace petroleum-based polymers in some applications [8,27,28]. Interest in nature-based biopolymers from the marine environment is increasing, as they avoid the use of land resources destined for crops. In this sense, oceans provide a wide variety of these renewable natural macromolecules (Figure 1), with adjustable physicochemical and structural characteristics and appealing biological properties. These biopolymers can be supplied by algae, fish, crustaceans, and molluscs, which are obtained by chemical and/or biological extraction treatments. Common marine proteins include collagen, and gelatine can be obtained from fish by-products. Moreover, common marine polysaccharides are chitin and chitosan, provided by crustaceans’ shells, and alginate, agar, and carrageenan, obtained from macroalgae [4,8,11]. Even so, the production of these compounds by marine organisms can be affected by the presence of microplastics in the oceans. The effects might include interruptions of biological processes, which compromise the health and ecological functions of the organisms. For instance, Li et al. [29] observed an increase in the soluble protein and polysaccharides content of the microalgae *Scenedesmus quadricauda* in response to an increase in the concentration of nanoplastics. Conversely, Dianratri et al. [30] found that microplastics impact *Spirulina* sp. growth, decreasing its polysaccharide content. Additionally, Shiu et al. [31] demonstrate that exposure to nanoplastics induced stress to marine phytoplankton cells, altering the secretion of exopolymeric substances. Specifically, these authors found that the protein/polysaccharides ratios in these exopolymeric substances were increased.

**Figure 2:**
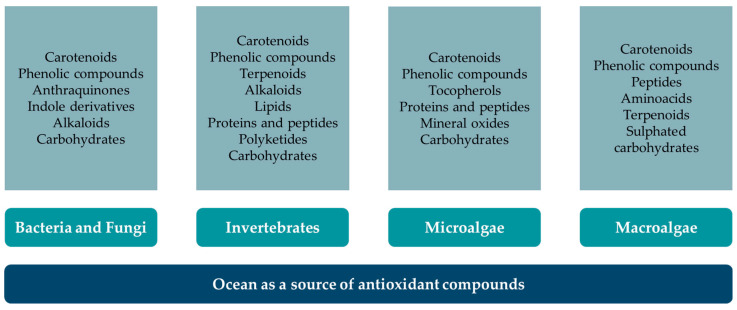
Antioxidant compounds from oceans and their sources.

As pressure grows to find natural and sustainable alternatives to synthetic additives, the marine environment stands out as a promising and largely underexploited source of high-value antioxidants. Marine organisms have evolved unique defence mechanisms to cope with harsh environmental conditions, leading to the production of potent antioxidant compounds (Figure 2). Seaweeds, for example, are rich in phlorotannins, fucoxanthin, and ascorbic acid, which exhibit strong radical scavenging and anti-inflammatory properties [32]. Marine invertebrates such as sponges and molluscs produce bioactive peptides and polyphenols with antioxidant activity [33,34], while marine bacteria and microalgae offer a sustainable source of carotenoids like astaxanthin and β-carotene [35]. These compounds have demonstrated significant potential in preventing oxidative stress-related diseases and are increasingly being explored for use in nutraceuticals, cosmeceuticals, and functional foods. However, once more, the presence of microplastics in marine environments might affect the production of antioxidants by marine organisms. On the one hand, Li et al. [29] demonstrated that nanoplastics activated antioxidant enzyme activities in *Scenedesmus quadricauda*. On the other hand, Choi et al. [36] found that microplastics induced intracellular reactive oxygen species generation in *Tigriopus japonicus*. In summary, microplastics impact the production of biomolecules by marine organisms, affecting biological processes in different ways. Consequently, further research is needed to fully understand these interactions and their consequences in the long term.

This review highlights the main biopolymers and bioactive compounds of marine origin, exploring their sources, extraction techniques, and main applications.

## 2. Methodology

The methodology for the literature selection was designed to ensure a comprehensive and systematic review aligned with the objectives of this study. As such, the first step was the conceptualization and the outlining of the review, considering all the subjects that needed to be addressed and the connections between them. As a result, a list of keywords was selected and searched on Google Scholar to capture a broad range of sources, such as primary articles, literature reviews, and applied studies in multiple fields (e.g., marine biotechnology, food science, health, and environment). The time frame of the publications was set to the last 20 years (2005–2025), with special emphasis on publications from the last five years, as an attempt to reflect recent developments and current knowledge in the field. Studies were included based on their relevance to the research question, methodological approach, and the availability of full articles in English.

The following keywords were combined in different manners and used for our literature search: Ocean/sea; Blue/sea bioeconomy; Plastic pollution; Sustainable development; Marine Organisms/resources; Red, brown and green macroalgae/seaweeds; Microalgae; Fish; Molluscs; Crustaceans; Marine by-products; Marine (sources of) biopolymers; Collagen; Gelatine; Hyaluronic acid; Marine Glycogen; Chitin; Chitosan; Microalgae; Exopolysaccharides; Carrageenan; Agar; Funori; Alginate; Fucoidan; Laminarin; Ulvan; Marine biopolymers extraction technologies; conventional extraction technologies; green extraction technologies; Ultrasound-assisted extraction; Microwave-assisted extraction; Hydrothermal extraction; Pulsed electric fields; Enzyme-assisted extraction; Marine polymers applications; Edible coatings; Marine (sources of) antioxidants; Sulphated polysaccharides; Bioactive peptides; Polyphenolic compounds; Flavonoids; Phlorotannins; Organic acids; Carotenoids; Marine antioxidants extraction technologies; and Marine antioxidants applications.

All identified records were imported into Zotero for de-duplication and screening. The titles and abstracts were reviewed to assess eligibility.

## 3. Marine Proteins

The most common marine proteins are collagen and gelatine, which are known to have a biodegradable nature and to be effective at forming gels, films, and coatings with advantageous characteristics like good oxygen and carbon dioxide barrier properties, UV light barrier, and transparency. Additionally, the structural and functional properties of these proteins make them useful in pharmaceutical, biomedical, leather, cosmetics, and tissue engineering industries [1,11]. Moreover, marine sources of collagen/gelatine show clear advantages over their mammalian counterparts, since there are no reports of possible transmissible diseases and they do not have religious/social limitation uses [37]. Table 1 summarizes marine sources, structure, extraction methods, applications, and main advantages of collagen and gelatine of marine origin.

### 3.1. Collagen

Collagen is a protein found in marine processing by-products, surimi production waste, squid skin, marine sponges, and jellyfish. It is mostly located in fibrous tissues like bones, cartilage, and skins, which are discharged and underused. Thus, this undervalued biomass has the potential to be exploited as a low-cost and eco-friendly collagen source [37,38].

Collagen molecules are formed by three peptide chains wound in a triple-helix structure, with a primary repeating tripeptide sequence of glycine-X-Y, where X and Y can be any amino acid but are mostly proline and hydroxyproline [39]. Nevertheless, there are some differences between the structure and chemical composition of marine and mammalian collagen. Marine collagen is less cross-linked than its mammalian counterpart and has lower contents of hydroxyproline, histidine, and tyrosine but a higher content of methionine. Consequently, marine collagen is less resistant to high temperatures, is more soluble, and has lower mechanical strength [37,40,41].

This protein presents high biocompatibility (low antigenicity) and excellent biodegradability, making it a promising candidate for various applications. It is applied in drug delivery, tissue engineering, dentistry, control bleeding, and cosmetics. Collagen can also be used in the food industry as a food additive or in packaging. However, no data about these applications are available for collagen of marine origin. There is only information about bovine, porcine, ovine, and duck feet collagen for food uses. Moreover, marine collagen can be used to produce gelatine by partial hydrolysis (in acidic or alkaline environment) [4,37].

### 3.2. Gelatine

Gelatine, a protein with a one-chain structure of 2–200 kDa, stands out for its versatility in different industries such as food and pharmaceutical [37]. In food, it is considered a “clean-label” product, since it does not contain and is not made of genetically modified organisms, is not chemically changed, is not a food additive with an associated E number, is generally recognized as safe (GRAS), and does not cause any known allergies [39]. Additionally, gelatine presents a film-forming ability and has been applied in active packaging strategies, since it reduces oxygen and oil transport and protects food from light and drying. These characteristics help reduce oxidation events, preserve flavor, and improve color stability, taste, and aroma of foods [42,43]. Its hydrophilic nature results in low moisture barrier properties. Gelatine from marine sources is a possible alternative to bovine gelatine and is currently used in gel-encapsulated drugs and in refrigerated and frozen food products. However, it has some constraints when compared to its mammalian counterpart. Depending on gelatine sources (i.e., animal species), the amino acid composition of this protein may vary significantly. Thus, caution needs to be taken, since fish alternatives have a lower melting temperature and strength, and may possess different odor and color as well as techno-functional and film-forming properties [25].

### 3.3. Protein Extraction Methods

Table 2 summarizes marine protein extraction methods and their recovery efficiency, with some examples of extraction yields obtained for the various extraction methods in different species.

The extraction/isolation of collagen from marine species is performed in three steps: preparation, extraction, and recovery. The preparation is initiated with cleaning, separating animal parts, and size reduction, which are essential to facilitate subsequent treatments. Secondly, the samples are treated with sodium hydroxide (NaOH) and ethanol to remove non-collagenous proteins, fats, and pigments. In samples composed of cartilage, bones, and scales, a demineralization with hydrochloric acid (HCl) or ethylenediaminetetraacetic acid (EDTA) is performed to increase the extraction yield. The extraction itself can be of two types: extraction of acid-solubilized collagen or pepsin-solubilized collagen, both being performed at 4 °C. Then, the collagen is recovered through precipitation with sodium chloride (2.3–2.6 M NaCl), centrifuged, dissolved in 0.5 M acetic acid, dialyzed, and finally freeze-dried [37].

Alternatively, greener extraction methods are being studied as complementary strategies to obtain collagen from marine sources. As such, ultrasound-assisted extraction (UAE), microwave-assisted extraction (MAE), and high-intensity pulsed electric fields (PEF) have emerged as promising alternatives to traditional collagen extraction methods. These methods offer advantages like reduced extraction time and increased extraction yields. Nonetheless, their use is still limited to laboratory or pilot scales. UAE is usually employed together with chemical or enzymatic extraction methodologies, facilitating mass transfer, which allows for higher extraction yields. Sonication disrupts the collagen structure, opening the fibrils, which facilitate acid and enzymatic treatment, reducing the extraction time of conventional methods. Nevertheless, the ultrasound application might disrupt the hydrogen bonds between the collagen chain, affecting the structural integrity of collagen [44,59]. Similarly to UAE, MAE isused as complementary treatment, usually followed by enzymatic hydrolysis. MAE improves the hydrolysis; however, overheating might occur and result in structure degradation [44].

Fish gelatine is obtained when the triple-helix structure of collagen is broken into single-strand molecules. For that purpose, a partial hydrolysis (acidic, alkaline, or enzymatic) is performed, followed by thermal treatment [4,37].

The application of novel technologies such as UAE, MAE, and high-pressure processing (HPP) has great potential to obtain marine gelatine but may not be sufficient for collagen hydrolysis. The combination of these methods with conventional technologies has proven effective and achieved promising results. Still, more investigation is needed to optimize the methodology conditions to obtain high-quality gelatine [57].

## 4. Marine Polysaccharides

Among the main marine biopolymers, polysaccharides can be easily isolated at low costs and have favorable functional properties, since their molecules interact through hydrogen bonds, forming a continuous network with good mechanical properties. Moreover, polysaccharides from marine organisms present various health benefits, such as antioxidant activity, which makes them attractive for the pharmaceutical industry [60]. Marine polysaccharides comprise animal-based polymers such as hyaluronic acid, extracted from soft connective tissues of fishes, glycogen, widely present as a storage polysaccharide in many marine organisms, and chitin and chitosan, which are extracted from residues like mussel and oyster shell, crustaceans (prawn and crab), and fish scale (pang and silver fishes) (Table 3). Alternatively, marine polysaccharides can be obtained from micro- and macroalgae (Table 4). Exopolysaccharides can be extracted from microalgae. We can find carrageenan, agar, xylogalactans, sulphated galactans, xylans, funori, porphyrin, and floridean starch in red macroalgae; alginates, laminarans, and fucoidans in brown macroalgae; and ulvans and cellulose mostly in green macroalgae [4,61,62,63,64].

### 4.1. Marine Animal Polysaccharides

Table 3 presents marine sources, structure, extraction methods, applications, and the main advantages of marine animal polysaccharides.

#### 4.1.1. Hyaluronic Acid

Hyaluronic acid can be found in cartilage and vitreous humour of fish. It is an anionic, highly hydrophilic, non-sulphated polysaccharide. It is made up of D-guluronic acid and N-acetyl-D-glucosamine units, linked via 1→4 and 1→3 bonds. This polymer exhibits a random coil structure that entangles at high molecular weights (up to 20,000 kDa) to form viscoelastic gels with lubricant properties [65]. These characteristics, together with its nonimmunogenic nature, high biocompatibility, and biodegradability, make hyaluronic acid useful in medical and biological applications like ophthalmology, tissue engineering, dermatology, cosmetics, and treatment of osteoarthritis [4]. This polysaccharide is usually obtained by hydrothermal extraction, followed by precipitation with ethanol [66].

#### 4.1.2. Glycogen

Glycogen is a polysaccharide present in animals and fungi across numerous taxa. It is found in many marine organisms like molluscs, fish, bacteria, invertebrates, and crustaceans, where it is an important precursor for chitin synthesis. Its structure consists of α-(1→4) D-linked glucose with varied branching degrees, and its molecular weight considerably varies depending on its source. Glycogen is a glucose supply that constitutes a short-term energy reservoir. As such, the amount of stored glycogen varies depending on species, food availability, and environmental conditions [29]. Therefore, glycogen levels tend to decrease when food is scarce, and the organisms need to utilize their stored energy to thrive. Conversely, when the environmental conditions are favorable, glycogen storage takes place, and a high glycogen content can promote gonad development and gametogenesis [29,67,68,69]. This polysaccharide participates in stress resistance and antioxidant defense mechanisms [29]. Glycogen also displays biological activity, including anti-inflammatory activity, and shows prebiotic potential, being fermentable by gut microbiota [67,70]. Thus, the application of marine glycogen in functional foods and nutraceuticals, for high energy applications, including sports and medical nutrition, shows potential [67]. Additionally, glycogen content is an oyster quality parameter related to its texture [29].

#### 4.1.3. Chitin

Chitin, the second-most abundant biopolymer in the biosphere, is a white, inelastic, hard, and nitrogenous linear polysaccharide composed of N-acetylglucosamine units linked via β-(1→4) bonds. It has a high molecular weight and is insoluble in water and in most organic solvents due to the presence of hydrogen bonds [71,72]. This polysaccharide is present in arthropods (shrimp, lobster, crab, insects) and molluscs (squid pen), and is also synthesized by some unicellular eukaryotic organisms like ciliates, cnidesporida, rhizopoda, diatoms, yeast, and fungi [4]. In nature, chitin exists as ordered crystalline microfibrils, closely associated with proteins, inorganic materials (mainly calcium carbonate), and lipids. Consequently, after the usual washing, drying, and grinding of the raw material, the extraction of chitin implies the use of HCl for demineralization, followed by a deproteinization with NaOH [73].

As a result of its abundance and structure, chitin shows unique physicochemical and biological properties like biocompatibility, antimicrobial activity, and biodegradability, which make it an attractive biopolymer for pharmaceutical and biochemical applications. Moreover, it is becoming one of the most important raw materials for the fabrication of emergent sustainable-based polymers [62]. Chitin is used in functional materials for environmental depollution as a flocculating agent to purify drinking water, to clean swimming pools, and to treat industrial wastewater, including nuclear wastewater. Furthermore, this polysaccharide is used in the food industry, in dietary supplements to address joint pain, and in cosmetics and toiletries as a moisturizing agent. It is also applied in the medical field to prepare suture threads; it is also blended with other polymers for drug delivery and tissue engineering. Finally, chitin is used in agriculture as a soil conditioner, in chemicals and pesticides [4,62,74]. Nonetheless, chitin is commonly converted into chitosan by partial deacetylation (above 50%) in highly alkaline environments at high temperatures or, in the presence of chitin, deacetylase enzymes [11].

#### 4.1.4. Chitosan

Chitosan is the N-deacetylated form of chitin, which, due to the presence of free amino groups, is soluble in aqueous acids such as acetic and lactic acids at low pH values. This abundant polysaccharide is cationic, colorless, can bind lipids, presents chelating ability for many metal ions, produces highly viscous solutions, and is inherently antimicrobial [71,72,75]. Due to its unique properties, chitosan has a wide range of applications and has been approved by the Food and Drug Administration (FDA) for certain food applications, such as edible film production. This polymer produces films with high mechanical strength and good barrier capacity to oxygen, thus being able to protect foods from deterioration [72]. It is also used as a preservative, an antioxidant agent, and an anti-cholesterol agent in the food industry [76,77]. Additionally, chitosan can be applied in water treatment and purification due to its ability to remove metals and pigments through adsorption. It is also used in agriculture as a coating for seeds and fertilizers, and in the pulp and paper industry for surface treatment and making adhesive paper. In cosmetics, chitosan is incorporated into body creams and lotions. Moreover, it has been approved by the FDA as a wound-healing agent and is utilized in artificial tissue reconstitution as well as in the pharmaceutical industry for the development of drug delivery systems [11,74,78].

### 4.2. Macro- and Microalgae Polysaccharides

Table 4 presents the marine sources, structure, extraction methods, applications, and main advantages of marine algae polysaccharides.

Microalgae exopolysaccharides (EPSs) are extracellular polysaccharides that can be excreted by microalgae in the environment around them or remain linked to the cell walls of these organisms, being easily isolated by ethanol precipitation [79]. These compounds are mainly heteropolysaccharides, and their composition varies with species, strains, and cultivation conditions like temperature, salinity, irradiance, and the availability of nutrients [80]. Generally, they are composed of xylose, glucose, and galactose, and considerable amounts of monosaccharides, such as fucose, methylated sugars, rhamnose, and iduronic acids. In addition, they can contain non-sugar substituents like pyruvate, proteins, and sulphate. Most microalgal EPS have high molecular weight and are characterized by a fluid-dynamic behavior that results in the production of highly viscous solutions at relatively low polymer concentrations [63,81]. Consequently, they are promising as thickening agents for food applications and can form biodegradable films. These compounds also show antioxidant, anti-inflammatory, and antimicrobial properties, which make them promising compounds for the pharmaceutical field. Currently, they are being applied in the cosmetics industry [80,81].

Marine macroalgae are highly available and sustainable, present high growth rates, high photosynthetic productivity, immense potential for carbon dioxide fixation, and high amounts of polysaccharides (generally, 50% of the seaweed dry weight) [82].

Macroalgae polysaccharides exist as cell wall polysaccharides, storage polysaccharides, and mucopolysaccharides, and are gaining a lot of attention in various applications, from food and energy to products like paper and plastic [83]. Seaweed-derived biopolymers are biodegradable, non-toxic, and have the ability to form films with unique physical, mechanical, thermal, and antioxidant properties, making them excellent candidates to develop safe packaging for foods and pharmaceuticals [84,85]. Despite forming films with good oxygen vapor barrier properties and being impermeable to oils and fats, they are soluble in water and show low tensile strength [86]. Nonetheless, these biopolymers have also been incorporated into food products as thickening agents, texture modifiers, stabilizers, and binders of ingredients, due to their water-binding capacity, gelation, and formation of emulsions and foams [87]. Even so, the industrial use of macroalgae is mainly focused on the extraction of phycocolloids (e.g., alginate, agar, and carrageenan) and bioactive compounds for direct food, cosmetic, and pharmaceutical applications [10].

#### 4.2.1. Red Macroalgae

Red macroalgae are widely used in various applications such as food, agriculture, cosmetics, and biomedical sciences. In addition, these macroalgae are known as rich sources of polysaccharides like carrageenan and agar, two anionic sulphated galactans widely used in the phycocolloid industry.

##### Carrageenan

Carrageenan is a polysaccharide with a high molecular weight that can be found in some red macroalgae genera like *Chondrus*, *Ahnfeltiopsis*, *Sarcodiotheca, Kappaphycus*, *Gigartina*, *Eucheuma,* and *Hyonea* [88]. It is a hydrophilic and anionic hydrocolloid that belongs to the class of linear sulphated galactans. It makes up 50% of the dry weight of these macroalgae. Its composition is mainly made up of potassium, sodium, magnesium, and calcium salts or sulphated esters of galactose and 3,6-anhydro-galactose copolymers, linked by α-1,3 and β-1,4-glycosidic bonds. Nonetheless, other carbohydrate residues may be present, such as uronic acids, glucose, xylose, and some substituents such as the pyruvate group and methyl ethers [82,83].

This polymer can be classified into three types according to the degree of sulphation and the position at which the sulphate group is connected to the galactose unit, namely kappa (k)-carrageenan, iota (ԏ)-carrageenan, and lambda (λ)-carrageenan. All three are water-soluble; however, their solubility is influenced by the temperature, pH, and ionic strength of the medium, as well as by the presence of cations [84]. (k)-carrageenan is composed of alternating 3-linked β-D-galactose-4-sulphate and 4-linked 6-anhydro-α-galactopyranose, having one sulphate group/disaccharide repeating unit. This carrageenan forms hard, strong, and brittle gels that are stable at room temperature. (ԏ)-carrageenan has two sulphate groups/disaccharide repeating unit, resulting in soft and weak gels, which are stable at room temperature. These two types exhibit a gelling ability in the presence of cations, which is influenced by the concentration and the nature of cations in the solution and by the polysaccharide concentration. (λ)-carrageenan possesses three sulphate groups/disaccharide unit, is only cold-water soluble, and is unable to form gels, exhibiting a random coil conformation at all temperatures [83,84]. Still, (λ)-carrageenan has thickening properties and is used for that purpose in dairy products [86].

Carrageenans are extensively used in pharmaceuticals, cosmetics, food, printing, painting, and textile formulations. These polymers are used in shampoos and hand lotion formulations and are applied as stabilizers in toothpaste preparations. They are also applied in wound dressings and as excipients in controlled drug release delivery systems [1,88]. Nonetheless, they can exhibit adverse effects toward blood coagulation and immune systems. These marine polysaccharides are also broadly applied in the food industry, which contributes to 70% of its consumption [11,83]. Carrageenan is used for beer clarification through precipitation with proteins, to prevent separation of whey in cottage cheese and as a fat substituent in milk products. It is applied as stabilizer and binder in the meat manufacturing industries for the production of low-calorie sandwiches, patties, and sausages, where it improves moisture retention and restore tenderness in low-fat processed meat. In this industry, carrageenan is also used as an oxygen barrier to retard lipid oxidation. Furthermore, these polymers are applied in the baking industry due to their excellent functional properties—efficiently bind water while thickening, stabilizing, and improving the appearance of foods. Additionally, they are used in beverages, pet food, infant formula, jam, syrups, sauces, and salad dressings, since carrageenans are cold soluble thickeners. Finally, these polysaccharides can be utilized to produce protective coatings; however, they show low water vapour barrier and low mechanical properties. Carrageenans are also known for having biological activities that include anti-inflammatory, anti-thrombotic, antioxidant, anti-viral, and cholesterol-lowering effects [83,87,89].

These polysaccharides can be isolated via alkaline extraction (with sodium or potassium hydroxide), followed by precipitation with ethanol. However, the extraction has some drawbacks, since the process is time-, energy-, and water-consuming [11,90].

##### Agar

Agar is an unbranched polysaccharide that forms a supporting structure in the cell walls of marine red macroalgae, namely *Gelidium* sp., *Gracilaria* sp. and *Pteroclodia* sp. [7]. This polymer can be extracted with hot water after an alkaline pre-treatment with NaOH [90]. It is a high-molecular-weight polymer composed of two groups of polysaccharides—agaropectin and agarose. The first one, agaropectin, is branched and contains several anionic groups such as sulphate, pyruvate, and glycuronate, which makes it a non-gelling fraction that is usually removed in the industrial production of agar. Conversely, agarose is a neutral linear polysaccharide constituted of repetitive units of D-galactose and 3,6-anhydro-L-galactose, linked by altering α-(1→3) and β-(1→4) glycosidic bonds [4,61]. Agarose, oppositely to agaropectin, has gelling capacity, which makes it useful in skin care, medicine, and pharmaceutical applications, and suitable for films and coatings preparation [86]. In concentrations ranging from 1.5 to 4% m/v, agar forms films with a thermoplastic behavior without any environmental impact, since, due to their natural and renewable background, they can be easily compostable or biodegradable. The produced films are transparent, heat-sealable, biologically inert, stable at low pH, and thermo-reversible. However, they are brittle, have poor mechanical properties, and show a high retraction ratio due to syneresis of agar gel during drying [9,61,82].

Additionally, agar has other important applications, being widely used in the food industry as a thickener, stabilizer, and emulsifier agent in gel-based food products such as desserts, jams, jellies, and bakery products, and is the vegetarian substitute of gelatine [87]. Agar also has applications in pharmaceutical and biotechnology studies, since it can act as a good biomedical impression material due to its thermo-reversible properties. It is also used for biochemical purposes like chromatography, agarose gel electrophoresis, and as culture media for microbiology, because it is not easy to metabolize but has good gel firmness, elasticity, stability, and clarity. In the medicinal field, it can be used to obtain monoclonal antibodies, alkaloids, interferons, and steroids, and act as a laxative and anticoagulant [4,11,84].

##### Funori

Funori is a mucilage polysaccharide from a family of red macroalgae (*Gloiopeltis* sp.) that is typically found in the Pacific coasts of Japan, Korea, southern China, and North America. It can be extracted with water, followed by precipitation with acetone and diethyl ether, which allows its recovery [91]. Funori is a heterogeneous polysaccharide, similar to agar but with a higher sulphate content, which makes its chemical and structural qualities close to those of carrageenans [92]. It is characterized by repeating units of β-D-galactose-6-sulphate and 3,6-anhydro-α-L-galactose [93]. As a result of its structure, funori solutions remain liquid even at room temperature. However, they may form gels in contact with some salts like potassium chloride [91].

This polysaccharide is widely used in Japan as food, medicine, thickening agent, adhesive, conservation material, and in hygiene and cosmetics formulations. Its main application is in conservation specialties like paper, textile, paintings, and wood, where it does not change the appearance or the mechanical properties of the materials. This polymer is very useful in artworks because it does not interact with the painted surface, produce any shine after application and drying, or change the color or tone of the consolidated surface. It is transparent, non-toxic, remains stable, and is easily removed after aging. Finally, it is also applied to repair silver and gold leaves and mica [1,94,95].

#### 4.2.2. Brown Macroalgae

Brown macroalgae are rich sources of dietary fibres (undigestible polysaccharides). They contain large amounts of polysaccharides, mainly alginate, fucoidan, and laminarin, that can be applied in food products as functional components and can be used as potential feed additives [96].

##### Alginate

Alginate is an anionic linear polysaccharide that is soluble in water and provides flexibility and strength to the cell walls of brown macroalgae, where it is mainly found in amounts up to 40% of dry weight [84]. Additionally, this polymer can be synthesized by some bacteria such as *Pseudomonas aeruginosa* and *Azotobacter vinelandii* [4]. Prior to the extraction of alginate with sodium carbonate (Na_2_CO_3_), macroalgae are pre-treated with HCl, and finally, this polymer is precipitated with ethanol [90].

The structure of this polysaccharide comprises monomer units of β-D-mannuronic acid (M) and α-L-guluronic acid (G), joined by 1,4 linkages. Depending on the ratio of these monomers, alginate produces gels with different characteristics. For higher G content or high molecular weights, stronger and more brittle gels are obtained. On the contrary, higher amounts of M lead to more flexible gels. Moreover, in the presence of calcium cations (Ca^2+^), alginate forms rigid and stable gels, with higher tensile strength and elongation and reduced opacity [8,86].

The intrinsic properties of this polysaccharide, together with its biodegradability, biocompatibility, low toxicity, and low extraction and purification costs, make it a promising biopolymer for a wide variety of applications [4]. Alginate is used in textile printing, in binders for fish feed, to immobilize biocatalysts and release agents, in heavy metal absorption, in pharmaceutical and medicine, for the encapsulation of drugs and drug delivery, and in wound healing materials. In the food industry, it is widely used for limiting the dehydration of meat, fish, and fruits as emulsifiers and as stabilizers in ice creams; in beverages, it is used as a thickening, gel-forming, and colloidal stabilizing agent [89]. Moreover, this polysaccharide can be used as a coating material and in bioplastics production, where the addition of calcium provides more stability and resistance to the membranes. The resulting films are impermeable to oils and fats, provide good oxygen barriers, and are uniform and transparent [8,11,97].

##### Fucoidan

Fucoidan is a branched sulphated polysaccharide that can be found in the cell wall of brown seaweeds and in marine invertebrates like echinoderms [98]. In brown seaweeds, along with alginate and cellulose, fucoidan represents the major component of the cell walls, constituting 2–20% of their dry weight. It is usually obtained by hydrothermal or acidic extraction, and salts like CaCl_2_ are frequently used to increase the fucoidan’s purity [64,82,99].

Its structure and composition vary depending on algae species, harvesting season, geographical location, and extraction procedures. As such, variations in sugar content, glycosidic linkages, molecular weight, branching sites, and sulphate ester pattern and content are observed [100,101]. Nonetheless, it is composed of a sulphated fucose backbone, but also contains other monomers like uronic acid, xylose, galactose, mannose, rhamnose, glucose, and arabinose [102,103]. Monomeric units of fucoidan are linked via α-(1-2) or α-(1-3) bonds, and its molecular weight ranges from 13 to 1600 kDa. Some proteins and minerals like calcium, magnesium, manganese, copper, potassium, sodium, selenium, and zinc can also be found in the fucoidan structure [98,101].

Historically, fucoidan has been employed in conventional Chinese medicine for treating various conditions, from cardiovascular diseases to ulcers, renal problems, arteriosclerosis, asthma, and eczema. More recently, this polysaccharide has attracted the interest of pharmaceutical, food, and biomedical researchers because of its structural features and wide range of biological activities. Fucoidan has demonstrated anticoagulant, antioxidant, anti-inflammatory, anti-obesity, anti-allergic, and antimicrobial activities, being applied in nanomedicine, pharmacology, cosmeceuticals, nutraceuticals, and pharmaceuticals [99,100,101,104]. Additionally, this polymer has a strong ability to bind numerous compounds, and when mixed with oppositely charged polymers, it can produce gels, matrices, and films [98]. Moreover, fucoidan shows high stability under acidic and alkaline conditions and high thermal stability, which makes it ideal for food processing and pharmaceutical manufacturing. As such, its properties, which include bioactivity, biodegradability, and biocompatibility, make fucoidan an attractive candidate for tissue engineering, drug delivery, and technologies like 3D printing [98].

#### 4.2.3. Green Macroalgae

Green macroalgae are widely distributed and particularly common in areas with abundant light such as shallow waters. These macroalgae are remarkably diverse, and the main genera include *Ulva*, *Codium*, *Chaetomorpha*, and *Cladophora* [105]. Despite their importance, green macroalgae are the most underexplored phylum; thus, they still hold a remarkable potential for innovation. Recently, these macroalgae have been more studied due to their applications in different sectors including biorefinery operations, land-based integrated multitrophic aquaculture systems, biofuel, bioremediation, and high-value food products in modern cuisine [106]. Additionally, green macroalgae show interesting properties that make them potential candidates for the production of biodegradable films. They can be used for the extraction of polymers such as cellulose and other sulphated polysaccharides, like ulvan [82,84].

Among all macroalgae species, green macroalgae of the genus *Ulva* represent a significant portion of the global biomass. They are cosmopolitan, have high growth rates, thrive in diverse climate zones, and are suggested to be less sensitive to global warming. Consequently, *Ulva* are the most-studied green macroalgae as they can contribute to sustainable bioeconomy, and so ulvan is the most-studied polysaccharide from this phylum [22,107].

Ulvan is a sulphated heteropolysaccharide, constituting 9–36% of algal dry weight [107]. This polymer, similar to the other macroalgae polysaccharides, is extracted with hot water and then precipitated with ethanol [90]. It is an anionic polymer that structurally consists of disaccharide repeating units of D-glucuronic acid or L-iduronic acid linked to L-rhamnose-3-sulphate and ulvanbioses or xylose-2-sulphate linked to rhamnose-3-sulphate [82,106]. Its structural characteristics reflect on functional properties like antioxidant, anticoagulant, antihyperlipidemic, antimicrobial, anti-viral, and immunomodulatory activities. Consequently, ulvan is considered a promising bioactive compound for the development of various applications like wound dressings, tissue engineering, animal feed, cosmetics, and drug delivery systems [107].

Additionally, ulvan has high viscosity and gelling properties and film-forming ability, being able to form thermo-reversible films due to its polyanionic nature and the presence of hydrophilic (OH, COOH, SO_4_) and hydrophobic (CH_3_) functional groups. Ulvan can alternatively be mixed with other polymers such as chitosan, polyvinyl alcohol, and polyethylene oxide to improve the mechanical properties of the films [8,22].

### 4.3. Polysaccharide Extraction Methods

Marine-origin polysaccharides (from animal, micro-, and macroalgae) are readily and widely available, being usually obtained by solid–liquid extraction. Usually, these polymers are extracted in water at high temperatures. Yet, the scientific community is working on more efficient and eco-friendly approaches to minimize extraction time and energy and reduce solvent consumption [108,109]. Table 5 and Table 6 summarize extraction methods and their recovery efficiency, with some examples of extraction yields obtained for the various extraction methods of marine animal polysaccharides and algae polysaccharides, respectively.

The preparation of the raw material before the conventional polysaccharide extraction is the same for animals and micro- and macroalgae. Firstly, the raw material is collected and cleaned in running water to eliminate impurities. Secondly, it is dried in an oven and ground into powder to increase the surface/contact area. Then, some pre-treatments are usually performed to eliminate interfering substances like minerals, lipids, and proteins [109].

For chitin isolation, after grinding, the biomass goes through an acidic demineralization step, usually using HCl, and then deproteinization is performed in an alkaline solution, often with NaOH. Finally, the solution is washed with distilled water until neutrality is achieved, and the resulting solid product is dried in an oven until a constant weight is achieved. Alternatively, the deproteinization and demineralization steps can be performed using some bacteria strains. However, at the industrial level, chemical extraction is used because it is a low-cost method that can be used in large quantities. Chitosan can be obtained by the partial deacetylation of chitin in a highly alkaline environment at high temperatures or, alternatively, using chitin deacetylase enzymes [11,72].

Alternatively, chitin can be recovered through subcritical water extraction (SWE) or enzymatic extraction (EE). These methods enable effective removal of the protein fraction from the polymeric structure, reduce the need for solvents, and improve both extraction rate and yield. However, they remain too costly for large-scale industrial applications [108].

To obtain hyaluronic acid, a hydrothermal extraction is performed, followed by precipitation with ethanol. Instead, enzyme extraction can be employed to recover hyaluronic acid polysaccharides. Papain is the most used enzyme, yet it is a time-consuming method with high energy consumption. Thus, it can be coupled with extraction methods, like MAE, UAE, or PEF, to surpass these drawbacks [108].

**Figure 3:**
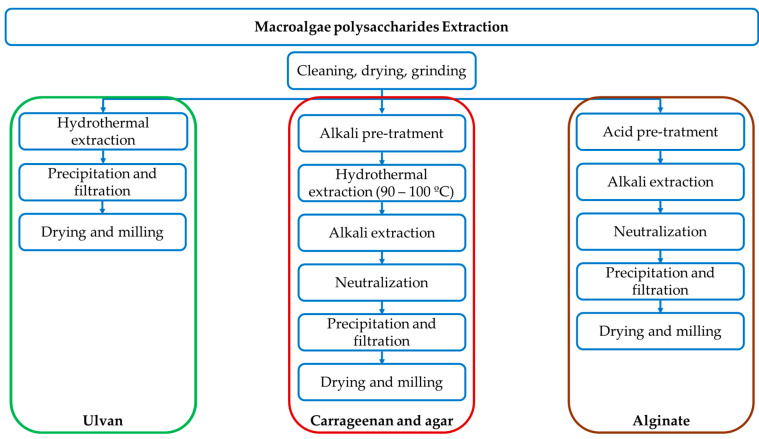
Schematic representation of the extraction processes of principal polysaccharides from macroalgae.

From microalgae, exopolysaccharides can be easily isolated by ethanol precipitation. In macroalgae, depending on the target polysaccharides, an alkaline or acidic pre-treatment is followed by hydrothermal extraction (Figure 3). The extracted polysaccharides are crude mixtures with variable molecular weights, monosaccharide composition, and sulphate content. They are usually purified by ethanol precipitation, which removes low-molecular-weight impurities [109].

As with other polymers, greener technologies are emerging for the extraction of polysaccharides from macroalgae. For example, microwave-assisted extraction (MAE), ultrasound-assisted extraction (UAE), and enzyme-assisted extraction (EAE) have been studied as eco-friendly and time-saving methods that offer higher extraction yields [108].

## 5. Sources of Marine Antioxidants

Oceans have a set of unique conditions for the development of life, characterized by variations in pressure, salinity, lightning, temperature, and availability of nutrients. These conditions foster the production of a wide array of secondary metabolites and macromolecules with diverse biochemical and therapeutic potentials, like anti-inflammatory, antiproliferative, and antioxidant activity (Table 7) [137]. As such, the marine environment constitutes a goldmine of antioxidant compounds [138,139].

Antioxidants are molecules that, at low concentrations, can prevent the oxidation of a substrate, delaying the degradation of biomolecules. Consequently, antioxidants find applications in nutraceuticals, pharmaceuticals, medicinal, and cosmeceutical industries. They are widely applied in the treatment of some diseases and in foods as an effective way to minimize and prevent lipid oxidation, maintain nutritional quality, and prolong the shelf life of food products. Notwithstanding the antioxidant capacity of these molecules, their application is facing some challenges. The extraction yield and the bioactivity of the extracted compounds depend on the marine species, the extraction and purification technologies, and the climatic conditions under which the biota was grown [34].

In the marine environment, a plethora of organisms, such as macroalgae, sponges, microalgae, crustaceans, fish, ascidians, bryozoans, lichen, bacteria, sea cucumbers, and fungi, are rich sources of antioxidant compounds like sulphated polysaccharides, bioactive peptides, and polyphenolic compounds, such as flavonoids, phlorotannins, and organic acids [148].

### 5.1. Bacteria and Fungi

Oceans and seas account for about 90% of the living mass of our planet, and microorganisms, which include bacteria and fungi, are broadly represented. These microorganisms produce a wide range of bioactive compounds like carotenoids, phenolics, anthraquinones, indole derivatives, alkaloids, and carbohydrates, which exert antimicrobial, antiproliferative, anti-inflammatory, and antioxidant activities. Due to their unique properties, these antioxidants might be used in a variety of fields, including food, cosmeceuticals, and pharmaceuticals [147,149].

Marine bacteria produce carotenoids and exopolymers with strong antioxidant activity to protect themselves from the environmental conditions. These exopolymers are essentially made of polysaccharides (exopolysaccharides—EPS) and proteins, but may also include other molecules like lipids, DNA, and humic substances. They are used in varied applications like film development, gel arrangement, emulsification, absorption, thickening, and anticancer treatment, and show potential in the development of new natural drugs. Previous works suggest that bacterial growth, generally accompanied by the production of EPS, is a safe and low-cost way of obtaining noteworthy antioxidant compounds [34,149,150].

Marine fungi are a prolific source of structurally diverse bioactive metabolites with antioxidant capacity that can be extracted at low costs. These metabolites include phenolic compounds, anthraquinones, xanthones, carotenoids, and polysaccharides, and the last ones tend to have a simple monosaccharide composition and low molecular weight. Fungal polysaccharides, especially exopolysaccharides, are very effective antioxidants, rapidly produced, easy to purify, and do not require an extraction with organic solvents.

Marine fungi biosynthesize carotenoids, fat-soluble pigments that protect microorganisms against oxidative damage. Among the carotenoids extracted from marine fungi, astaxanthin is the strongest antioxidant due to its structure. In what concerns phenolics and their derivatives, marine fungi produce anthraquinones and xanthones, both with antioxidant capacity. Depending on the extraction target, different extraction and purification methods are employed. Commonly, a liquid–liquid extraction is applied, and the solvent is selected based on the polarity of the target antioxidant [147,151].

### 5.2. Invertebrates

Marine invertebrates exist in all ocean habitats, from shallow coral reefs to the deep sea, and play ecological roles, contributing to the diversity and functioning of marine ecosystems. They are a diverse group of organisms that include sponges, jellyfish, sea anemones, corals, bivalves, sea cucumbers, crustaceans, and molluscs, being rich sources of natural products with multiple applications. As a result of their growth conditions, marine invertebrates produce a plethora of primary and secondary metabolites such as polyketides, terpenoids, alkaloids, lipids, proteins, and polysaccharides, that exhibit highly relevant biological properties (antioxidant, anti-inflammatory, neuroprotective). Consequently, they find multiple industrial applications in the cosmetics, food, and aquaculture sectors [33,152,153].

Sea cucumbers are marine animals that have been widely used for food, cosmetics, and traditional medicine in Asian countries. These invertebrates are broadly known to be an excellent source of bioactive compounds with antioxidant activity, such as phenolics, carotenoids, polysaccharides, and peptides. In what concerns phenolic compounds, sea cucumbers have mainly phenolic acids and flavonoids like chlorogenic acid, gallic acid, p-coumaric acid, protocatechuic acid, ferulic acid, ellagic acid, cinnamic acid, catechin, rutin, quercetin, and pyrogallol. The polysaccharides are mainly represented by fucan and fucosylated chondroitin sulphate, two sulphated polysaccharides with antioxidant activity. The chain conformation, molecular weight, degree of sulphation, type of main sugar, and glycosidic bonds influence the antioxidant activity of these polymers. Apart from these compounds, sea cucumbers are also rich in carotenoids, especially astaxanthin and canthaxanthin, peptides, saponins, and cerebrosides, which also show antioxidant activity. Nevertheless, these compounds are presented at different concentrations and show distinct antioxidant activities between various animals, which might result from geographic location, food habits, harvesting times, species, and body part. Thus, it is important to understand the detailed chemical structures of the compounds, their mechanism of action, bioaccessibility, and bioavailability [154].

Crustaceans are natural sources of bioactive compounds with antioxidant properties, like chitin derivatives, bioactive peptides, and carotenoids. In what concerns chitin derivatives, such as chitooligosaccharides (COS), they are obtained by chemical or enzymatic hydrolysis of chitosan, showing a degree of polymerization up to 20 and an average molecular weight of 3900 Da. COS are polycationic polymers mainly comprising glucosamine units that are non-toxic and highly soluble. The antioxidant activity of COS is ruled by the presence of active hydroxyl and amino groups, which can react with free radicals, and varies with molecular weight and degree of deacetylation [155].

Bioactive peptides are generally obtained by enzymatic, chemical, or microbial hydrolysis, resulting in varying peptides, mostly with 2 to 20 amino acids. Usually, these peptides have excellent metal ion (Cu^2+^/Fe^2+^) chelating ability and antioxidant activity that varies with the degree of hydrolysis, molecular structure such as the length of the peptide chain, amino acid composition, hydrophobic/hydrophilic properties, and charge of amino acid [156]. These peptides exhibit high bioactivity, along with low immunogenicity. Some of them are currently undergoing clinical trials and are anticipated to contribute to the anticancer drug discovery pipeline. However, bioactive peptides present some concerns like instability, which can be overcome through some structural modifications like nanoencapsulation, allowing an improvement in their targeted delivery [137]. Finally, carotenoids might be extracted from shell wastes of crustaceans using solvents like acetone, methanol, and chloroform. The most common carotenoids in these organisms are fucoxanthin and astaxanthin, both with recognized antioxidant activity [157].

### 5.3. Microalgae

Microalgae are eukaryotic unicellular organisms that can accumulate bioactive metabolites which are widely used in the nutritional food, pharmaceutical, and cosmetics industries. Over the last few years, microalgae have represented a significant source of natural antioxidants and have drawn the attention of the markets as sources of vitamins, pigments, phenols, polysaccharides, proteins, mineral oxides, and essential fatty acids [34].

They are rich in water-soluble vitamins like vitamin C (ascorbic acid) and in liposoluble vitamins like vitamin E (tocopherols). In what concerns pigments, some microalgae produce marennine, a blue-green pigment, and phycobiliproteins—water-soluble pigments that participate in photosynthesis. In addition, they contain various carotenoids like β-carotene, astaxanthin, fucoxanthin, lutein, keraxanthin, zeaxanthin, and lycopene, fat-soluble pigments of great interest to the food industry [35,158]. The main phenolic compounds identified in these organisms are phloroglucinol and phenolic acids derived from hydroxybenzoic acid and hydroxycinnamic acid. Flavonoids can also be present, but at lower concentrations.

Additionally, microalgae have glutathione, a water-soluble tripeptide. All the described molecules have shown antioxidant activity. Moreover, certain microalgae produce specific antioxidant molecules—mycosporins-like amino acids, which are colorless and water-soluble compounds. Furthermore, these organisms have some features that make them attractive to the industry such as high biodiversity, photosynthetic yield, growth, productivity, and metabolic plasticity that can be optimized using culture conditions [145].

### 5.4. Macroalgae

Macroalgae or seaweeds are the base of the aquatic food web, contributing substantially to aquatic life; however, they remain underutilized. They stand out as rapidly growing organisms with no requirements for freshwater, arable land, fertilizers, or pesticides, and are usually available all year round, with some seasonal variations in growth. As such, seaweeds have become an attractive and sustainable source of structurally diverse natural compounds that often exhibit significant biological activities [159,160].

Currently, macroalgae are being explored as a rich source of extraordinarily potent marine antioxidants such as sulphated polysaccharides, phenolic compounds, pigments, amino acids, and vitamins that can be used for numerous applications. As mentioned above, these organisms are the most important source of non-animal sulphated polysaccharides, which comprise a complex group of macromolecules like fucoidan, carrageenan, and ulvan, that exhibit health-beneficial biological activities. Despite their nutritional features, sulphated polysaccharides are reported to have antioxidant activity, which is directly related to their structural features such as molecular weight, type of main sugar, glycosidic branching, sulphation position, and sulphation degree. For instance, low molecular weight polysaccharides have higher antioxidant activity because they can be incorporated into the cells and can donate protons more efficiently [34,88,151].

In what concerns phenolic compounds, marine macroalgae are rich sources of these secondary metabolites that include simple phenolic acids and polyphenols, flavonoids, and non-flavonoids. Halogenated phenolics, namely bromophenols, are also found in macroalgae, and they contain one or several phenolic rings connected to one or more bromine atoms [138]. Structurally, phenolic compounds are conjugated rings with hydroxyl groups that can scavenge and stabilize radicals. Thus, these compounds show pronounced antioxidant activity, which is related to the number and position of hydroxyl moieties, the substitution of aromatic rings, and the degree of polymerization. Usually, lower degrees of polymerization result in greater antioxidant activities [32,161].

Phlorotannins are a heterogeneous group of highly hydrophilic polyphenols only synthesized by brown algae with a wide range of molecular sizes (126–650 kDa). These compounds are formed by the polymerization of phloroglucinol and consist of eight interconnected aromatic phenyl rings with hydroxyl groups which make them potent antioxidant compounds. In addition to their antioxidant activity, phlorotannins contribute to other roles in macroalgae, namely chelating divalent metal ions, being integral structure constituents that bind with polysaccharides, proteins, and other biopolymers, and also by showing antimicrobial activity. Besides phenolic compounds, seaweeds also synthesize terpenoids, secondary metabolites composed of isoprene units. However, when compared to phenolics, terpenoids are less active as antioxidants [138,162].

Marine algae produce natural pigments with antioxidant activity like carotenoids, responsible for orange/red colors. Carotenoids are tetraterpenoids with a highly unsaturated structure, which makes them oxidize in place of other molecules; thus, they are efficient antioxidant compounds. Their antioxidant actions are based on their singlet oxygen quenching properties and their ability to trap free radicals, which mainly depends on the number of conjugated double bonds and carotenoid end groups. In addition, they are known to have anti-inflammatory and immune-boosting actions and to decrease the risk of chronic diseases in humans. In macroalgae, β-carotene, lutein, zeaxanthin, astaxanthin, neoxanthin, fucoxanthin, and violaxanthin are known to be among the major carotenoids encountered. Fucoxanthin is an oxygenated carotenoid that shows greater antioxidant activity than the other carotenoids due to the presence of conjugated double bonds. It is acknowledged as an efficient quencher of singlet oxygen and a radical scavenger, and it effectively inhibits the intracellular formation of oxygen radicals [32,155].

Moreover, macroalgae contain proteins of high nutritional value that could be used to obtain novel antioxidant peptides, since they are a rich source of aspartic acid, leucine, and glutamic acid. Actually, algae waste contains over 50% of protein, but are frequently used for animal feed, a low-economic-value product. As such, they could be valued and used as a source of these antioxidant peptides [159].

### 5.5. Extraction of Antioxidant Compounds from Marine Sources

Regarding the extraction of antioxidant compounds from marine organisms, different species, environmental conditions, and collection locations result in distinct compositional profiles and biological activities. In addition, factors such as the extraction methodology, type of solvent used, biomass-to-solvent ratio, and storage conditions (e.g., raw, freeze-dried, or air-dried; particle size; storage temperature, light, and humidity), as well as extraction temperature and time, all influence both the extraction yield and the antioxidant activity of the resulting compounds.

Prior to the extraction, there are some steps that always take place, apart from the selected extraction method. After harvesting, raw materials are washed to remove epiphytes, salt, and sand, and then they are commonly dried. The biomass can be ground to different particle sizes to increase the surface area. Lastly, the samples are stored, and ideally, to preserve the maximum antioxidant activity, they should be kept in the dark, at a maximum temperature of 40 °C, and without contact with oxygen. After these preparation steps, different methodologies can be applied to the extraction. Traditionally, a solid–liquid extraction is used, and it is influenced by the antioxidant compounds’ solubility in the solvent system, solid–liquid ratio, extraction time, and temperature. For polar antioxidant compounds, such as phenolic compounds, the recovery efficacy increases with the solvent polarity. Nonetheless, this method requires large amounts of organic solvents, and it simultaneously co-extracts interfering compounds. Thus, it is not a very selective method and is also not environmentally friendly. Therefore, some technologies with different extraction principles are emerging and being developed such as enzyme-assisted extraction, microwave-assisted extraction, ultrasound-assisted extraction, supercritical fluid extraction, and pressurized-liquid extraction [34].

Enzyme-assisted extraction makes use of several digestive enzymes (proteases and carbo-hydrases) to break down the macromolecules, allowing the solubilization of antioxidant compounds in the solvent that tends to be water or another “green” solvent such as ethanol. In the microwave and ultrasound-assisted extractions, the biomass is submerged in a solvent and then submitted to irradiation (of microwaves or ultrasounds). This irradiation disrupts biomolecules of the matrix, allowing the solvent to penetrate and to solubilize the antioxidant compounds [146].

In supercritical fluid extraction, supercritical carbon dioxide tends to be the selected solvent because it is non-toxic and relatively cheap. In this physical state, the solvent has a low viscosity, the surface tension is negligible, and there is a high diffusion rate. This method is very useful for the extraction of heat-sensitive compounds. When pressurized liquid extraction is followed, water is the solvent used, and it is pushed beyond its boiling point but remains liquid due to the elevated pressure. The high temperature increases the solubility of the compounds, facilitating the diffusion and solvent penetration. Despite the high temperature used, usually, the antioxidants do not oxidize during extraction, probably because they are protected from light and oxygen. Nevertheless, caramelization and Maillard reactions might occur in the samples due to the high temperatures [146].

After extraction, antioxidant compounds can be separated and purified in order to attain a qualitative and quantitative characterization of the extracts. For separation, chromatography is usually the selected methodology. In the purification step, different fractions of the extract are obtained based on solubility, charge, chemical affinity, or molecular weight. Finally, the antioxidant compounds can be identified using nuclear magnetic resonance (NMR) or high-performance liquid chromatography (HPLC) coupled with mass spectrometry (MS) [159].

## 6. Conclusions

Oceans are being increasingly recognized as engines of economic development. However, they remain underexplored, and their extraordinary diversity of underutilized resources holds remarkable potential for growth. In this context, marine macroalgae are highly adaptable, sustainable, and widely available organisms. They do not require freshwater, pesticides, or arable land to grow, making them environmentally advantageous. Moreover, they are rich sources of polysaccharides and antioxidant compounds. Red and brown macroalgae are already well-known sources of hydrocolloids, while green algae, despite also containing valuable polysaccharides, remain largely untapped for this purpose. This highlights the need to explore this abundant phylum as a natural source of polymers and antioxidants. These materials have promising applications in the food industry, particularly in the development of active edible films and coatings, aligned with a circular marine bioeconomy. Additionally, fish by-products should be viewed as valuable raw materials for the development of new products. Rich in proteins, they can also be used to produce biodegradable films and coatings. Other innovative uses should be explored to unlock the potential of these often-overlooked marine resources. Still, the use, transformation, and application of less-explored raw materials and by-products is of utmost importance, supporting not only the environment but also economic development. The use of these resources creates new value from previously wasted materials, which can be used as sources of bioactive compounds, proteins, and polysaccharides. These molecules not only enhance the structural properties of food products but also exhibit important biological activities, such as antioxidant and anti-inflammatory effects, making them promising candidates for nutraceutical and pharmaceutical development. Translational research aims to integrate these compounds across various industries including food, pharmaceuticals, and nutraceuticals. However, despite their promising therapeutic potential in pharmaceutical applications, more clinical studies and interdisciplinary research are essential to validate their efficacy and standardize their extraction, characterization, and application. The adoption of biorefinery systems and sustainable practices can further support the efficient utilization and commercialization of these marine bioactives.

## Figures and Tables

**Table 1:** Marine proteins—structure, extraction methods, applications, and the main advantages of collagen and gelatine of marine origin.

Polymer	Marine Sources	Features/Structure	Extraction	Application	Advantages
Collagen	Fish by-products	Three peptide chains wounded in a triple helix	Extraction of acid- or pepsin-solubilized collagen at 4 °C; precipitation with NaCl	Tissue engineering; regenerative medicine; dental applications; wound dressing;	High biocompatibility; excellent biodegradability
				food additive;	
				cosmetics; drug delivery; clinical analysis	
Gelatine	Marine collagen	One-chain structure; molecular weight: 2–200 kDa	Partial hydrolysis of collagen in acidic or alkaline environment;	Active packaging; gel-encapsulated drugs; refrigerated and frozen food systems	High versatility; coatings with good oxygen and oil barrier properties
			thermal treatment of the hydrolysed collagen		

**Table 2:** Marine protein extraction treatments and their recovery efficiencies. The examples of extraction yields were obtained using various methods in different species.

Marine Protein	Extraction Method	Recovery Efficiency	Examples	References
Collagen	Salt-solubilization extraction	Low–moderate	14.14% yield in *Thunnus obesus;*	[44]
			2.18% yield in *Acipenser schrenckii* cartilage	[45]
	Acid extraction	Moderate–high	13.5% yield in *Thunnus obesus* skin;	[46]
			61.26% yield in *Thunnus albacares* skin;	[47]
			27.04% yield in *Acipenser schrenckii* cartilage;	[45]
			43.62% yield in *Pelodiscus sinensis*	[48]
	Enzymatic extraction (Pepsin)	High	19.2% yield in sponge (*C. reniformis*);	[49]
			55.92% in *Acipenser schrenckii* cartilage	[45,50]
	Ultrasound-assisted extraction	High	50.75% yield in *Pelodiscus sinensis*	[48]
	Microwave-assisted extraction	High		[44]
Gelatine	Acidic hydrolysis	High	20.95% yield in black tilapia fish skins	[51]
	Alkaline hydrolysis	High	48.1% yield in carp scales	[52]
	Enzymatic hydrolysis	High	50.89% yield in *Cyprinus carpio* scales	[53]
	Ultrasound-assisted extraction	Low–high (need pre-treatment)	46.7% yield in *Hypophthalmichthys nobilis* scales	[54]
			5.33% yield in *Pangasius hypophthalmus* fish bon	[55]
	High-pressure processing	Low–high (need pre-treatment)	32% yield in *Oreochromis niloticus* skin	[56,57]
	Microwave-assisted extraction	Low (need pre-treatment)	0.82% in common carp scales and fin	[58]

**Table 3:** Marine animal polysaccharides—source, structure, extraction methods, applications, and the main advantages of hyaluronic acid, chitin, and chitosan from marine origin.

Polymer	Marine Source	Features/Structure	Extraction	Application	Advantages
Hyaluronic acid	Cartilage and vitreous humour of fish	Non-sulphated polysaccharide; random coil structure	Hydrothermal extraction; precipitation with ethanol	Ophthalmology; tissue engineering; dermatology cosmetics;	Non-immunogenic nature; high biocompatibility biodegradable
				treatment of osteoarthritis	
Glycogen	Molluscs, crustaceans, fish, bacteria, invertebrates	α-(1→4) D-linked glucose; varied branching degrees and molecular weight	Hydrothermal extraction or alkaline extraction; precipitation with ethanol	Functional foods; nutraceuticals	Anti-inflammatory
Chitin	Arthropods, molluscs	Nitrogenous linear polysaccharide; insoluble in water and most organic solvents	Demineralization with HCl deproteinization with NaOH	Fabrication of polymers; purification of water; cosmetics; drug delivery; tissue engineering; soil conditioner	High biocompatibility; antimicrobial activity; biodegradable
Chitosan		cationic polysaccharide; soluble in aqueous acids	Partial deacetylation of chitin in highly alkaline environments	Edible film production; food industry; water treatment and purification; pulps and paper industry; cosmetics; pharmaceutical industry	Antimicrobial activity; films with high mechanical strength

**Table 4:** Summary of marine algae polysaccharides—source, structure, extraction methods, applications, and main advantages.

Polymer	Marine Source	Features/Structure	Extraction	Application	Advantages
Exopolysaccharides	Microalgae	Heteropolysaccharides; composition varies with the species; fluid dynamic behavior	Precipitation with ethanol	Food industry; Biodegradable film production cosmetics	Antioxidant, anti-inflammatory, and antimicrobial activities
Carrageenan	Red macroalgae	Linear sulphated galactan; high molecular weight; anionic, hydrophilic	Alkaline extraction Precipitation with ethanol	Pharmaceuticals, cosmetics, food industry, printing, painting, textile; coatings production	Anti-inflammatory, antithrombotic, anticoagulant, anti-viral, and antioxidant activities
Agar	Red macroalgae	Unbranched polysaccharide with high molecular weight	Alkaline pre-treatment Hydrothermal extraction	Cosmetics, medicine, pharmaceuticals; coatings preparation microbiology food industry	Does not have environmental impact; biologically inert
Funori	Red macroalgae	Heterogeneous polysaccharide; remains liquid even at room temperature; may gel in contact with some salts	Hydro-extraction Precipitation with acetone and diethyl ether	Food, medicine, cosmetics conservation material in artworks repair of silver and gold leaf and mica	Does not change the appearance or mechanical properties of the materials
Alginate	Brown macroalgae	anionic linear polysaccharide; hydrosoluble	Acidic pre-treatment with HCl; extraction with Na_2_CO_3_; precipitation with ethanol	Textile printing; pharmaceutical industry; food industry; coatings production	Biodegradable, biocompatible, low toxicity, low extraction and purification costs
Fucoidan	Brown macroalgae	Branched sulphated polysaccharide Heterogeneous chemical structures	Acidic extraction	Nanomedicine; pharmacology Cosmeceuticals nutraceuticals	Antioxidant, anti-inflammatory, and antimicrobial activities
Laminaran	Brown macroalgae	Neutral linear polysaccharide with low molecular weight; soluble in aqueous media or organic solvents	Hydrothermal extraction	Nutraceuticals, pharmaceuticals, cosmeceuticals; drug delivery, tissue engineering; functional food	Antimicrobial, immune-modulatory, anti-inflammatory, anti-coagulant, and antioxidant properties
Ulvan	Green macroalgae	Sulphated anionic heteropolysaccharide	Hydrothermal extraction; precipitation with ethanol	Wound-dressing, tissue engineering, drug delivery systems; animal feed; cosmeceuticals; coatings production	Antioxidant, anticoagulant, antimicrobial, anti-viral, and immune-modulatory activities

**Table 5:** Marine animal polysaccharide extraction treatments and their recovery efficiencies, examples of extraction yields obtained for the various extraction methods in different species.

Marine Animal Polysaccharide	Extraction Method	Recovery Efficiency	Examples	References
Hyaluronic acid	Hydrothermal extraction	Low	6.35 mg/mL	[66,110]
Glycogen	No available data was found			
Chitin	Chemical extraction	High	35.07% yield in Pang scale	[62]
			35.03% yield in mussel shell	[62]
			60% yield in crab	[62]
			69.65% yield in oyster shell	[62]
	Subcritical water extraction	High	82% yield in Cephalothorax	[111]
	Enzymatic extraction	Low	19.33% yield in *Litopenaeus vannamei*	[112]
Chitosan	Alkaline deacetylation	High	10.54% yield in crab shell waste	[113]
			39.5% yield in green mussel shells	[114]
	Enzymatic deacetylation	Moderate	Not specified	[113]

**Table 6:** Marine algae polysaccharide extraction treatments and their recovery efficiencies, examples of extraction yields obtained for the various extraction methods in different species.

Marine Algae Polysaccharide	Extraction Method	Recovery Efficiency	Examples	References
Exopolysaccharides	Ethanol precipitation	Moderate	27.25% yield in *Tribonema minus*	[115]
Carrageenan	Chemical extraction	Moderate–high	67.86% yield in *Kappaphycopsis cottonii*	[116]
	Ultrasound-assisted extraction	Moderate	50–55% yield in *Turbinaria ornata*	[117]
	Subcritical water extraction	High	78.75% yield in *Kappaphycus alvarezii*	[118]
Agar	Chemical extraction	Moderate	20.5% yield in *Gracilaria gracilis*	[119]
	UAE + EE	Moderate	10.9–18.2% yield in *Gelidium sesquipedale*	[120]
Funori	Chemical extraction	High	35% yield in *Gloiopeltis furcata*	[121]
Alginate	Chemical extraction	High	18.47–24.31% yield in *Sargassum polycisteum*	[122]
			22.5% yield in *Saccharina latissima*	[123,124]
	Ultrasound-assisted extraction	High	28% yield in *Sargassum binderi*	[117]
	Enzymatic extraction	Moderate	8–12% yield in *Saccharina latissimi*	[123,125]
	Subcritical water extraction	High	27.21% yield in *Saccharina japonica*	[126]
Fucoidan	Chemical extraction	Moderate–high	3.81% yield in *Sargassum* sp.	[127]
			11.9% yield in *Ascophyllum nodosum*	[123,128]
	Microwave-assisted extraction	Low -moderate	18.22% yield in *Fucus vesiculosus*	[129]
			5.71% yield in *Ascophyllum nodosum*	[123,128]
	Ultrasound-assisted extraction	Low–moderate	4.56% yield in *Ascophyllum nodosum*	[123,128]
	Enzymatic extraction	Low	3.89% yield in *Ascophyllum nodosum*	[123,128]
	Subcritical water extraction	High	14.93% yield in *Saccharina japonica*	[126]
Laminaran	Chemical extraction	Moderate–high	22% yield in *Laminaria gurjanovae*	[130]
			43.57% yield *in Durvillaea potatorum*	[131,132]
	Ultrasound-assisted extraction	Low–moderate	5.82% yield in *Ascophyllum nodosum*	[132,133]
			6.24%yield in *Laminaria hyperborea*	[132,133]
Ulvan	Chemical extraction	High	15% yield in *Ulva papenfussii*	[107]
			21.68–32.67% yield in *Ulva lactuta*	[134]
			19.8% yield in *Ulva rigida*	[135]
	UAE + EE	High	30.14% yield in *Ulva lactuta*	[136]

**Table 7:** Marine antioxidant compounds—sources, biological activities, mechanism of antioxidant capacity, other functional properties, and applications.

Antioxidant Compounds	Marine Source	Biological Activities	Antioxidant Mechanism of Action	Functional Properties	Applications	References
Peptides	Microalgae Macroalgae Fish by-products Invertebrates	Antioxidant Antimicrobial Anti-inflammatory	Free radical scavenging Metal ion chelating	Emulsifying Foaming	Food industry Cosmetic industry Pharmaceutical industry	[34,139,140,141,142]
Amino acids	Macroalgae Cyanobacteria	Antioxidant Photoprotective capacity	Free radical scavenging		Cosmetic industry	[34,143]
Polysaccharides	Bacteria Fungi Invertebrates Microalgae Macroalgae	Antioxidant Antiviral Antibacterial Anti-inflammatory Immunomodulatory activity Anticoagulant	Free radical scavenging Proton donation Metal ion chelating	Thickening agents Emulsifying Gel forming capacity	Nutraceuticals Pharmaceuticals Functional foods	[34,109,138]
Polyphenolic compounds (e.g., phenolic acids, flavonoids, anthraquinones)	Bacteria Fungi Invertebrates Microalgae Macroalgae	Antioxidant Antiviral Antibacterial Antifungal Anti-inflammatory Immunostimulant	Free radical scavenging Singlet oxygen scavenging Chelating agents Proton donation Electron transfer	Flavour compounds	Food and Feed industries Pharmaceuticals Functional foods	[34,144,145,146,147]
Terpenoids (e.g., Carotenoids, tocopherol, terpenoids)	Bacteria Fungi Invertebrates Microalgae Macroalgae	Antioxidant Anti-inflammatory Immunostimulant	Free radical scavenging Singlet oxygen scavenging Chelating agents Proton donation Electron transfer	Natural pigments	Food industry Cosmetic industry Pharmaceutical industry	[34,139,145,147]

## Data Availability

The original contributions presented in the study are included in the article, further inquiries can be directed to the corresponding author.
